# Benefits of a 12-week psychotherapy course for people with Parkinson’s disease: a service improvement project

**DOI:** 10.1007/s00415-024-12867-3

**Published:** 2025-01-15

**Authors:** Andreas-Antonios Roussakis, Rima Hawkins, Cara Mackley, Paola Piccini

**Affiliations:** 1 Imperial College London, Hammersmith Hospital, Hammersmith Campus, ICTEM Building, Ground Floor, Du Cane Road, London, W12 0NN UK; 2 University of Roehampton, Grove House, Roehampton Lane, London, SW15 5PJ UK; 3 Tavistock Relationships, 10 New Street, London, EC2M 4TP UK; 4 Birkbeck, University of London, Malet Street, London, WC1E 7HX UK

**Keywords:** Parkinson’s disease, Psychotherapy, Counselling, Intervention

## Abstract

The psychological symptoms of Parkinson’s disease (PD) worsen the quality of life of patients and their partners. However, these non-motor symptoms (mainly anxiety and depression) remain undiagnosed and undertreated in PD. Here, we report the benefits of a 12-week intervention using talking therapy (psychotherapy and counselling). This work was conducted in a group of individuals (people living with PD, and partners) with mild-to-moderate anxiety and depression. We discuss our results in context and conclude that short-term talking therapy should be integrated into PD therapeutics.

## Introduction

PD is experienced differently by everyone but it is evident that several aspects of the disorder can feel very isolating [1, 2]. As with any other progressive disorder, living with PD is associated with mental health implications. Individuals with chronic disorders, including those with PD, may struggle to deal with uncomfortable feelings and have difficulty in discussing those with the people nearest to them from fear of over-burdening them, or a fear of being misunderstood. This can impact negatively on interactions with others contributing further to distress and feelings of isolation.

The psychopathology of anxiety, depression, apathy, psychosis, impulse control, and sleep disorders is attributed to dysregulation in the dopaminergic, serotonergic, noradrenergic, and GABA-ergic pathways, which are all altered in PD [3, 4]. Medicines for the motor symptoms, depression, and anxiety are targeting the exact same pathways in the PD brain, and psychotherapy as an intervention has proven to be effective [5–7]. The burden of non-motor symptoms has been coined in PD in a systematic way [8] with clearly defined psychological and sexual elements. Yet, psychotherapy and counselling are less commonly considered as standard of care in PD in the NHS environment, signalling that existing services need improvement.

Distressing feelings such as anger, grief, shame, guilt, disgust, and sadness can be overwhelming, if the individual has not been used to expressing those appropriately and consistently. Therapy allows individuals with distressing feelings as well as speech and language problems to express themselves freely, and safely, and to understand the reasons they feel a certain way. In addition, therapy in PD offers the opportunity to discuss taboo subjects (such as sex and bowel problems) as well as impulsive and compulsive behaviours (such as binge eating and hypersexuality) which are somewhat PD-specific and not necessarily discussed in detail in the NHS clinics.

This project was designed to offer emotional support to PD patients who were open to the concept of talking therapy. The aim was to demonstrate the benefits of short course therapy, bespoke for patients with PD, and to increase awareness among physicians and patients with PD across north-west London, and secondarily, to generate interest for research in the wider PD community.

## Methods

The project was addressed to PD patients seen at NHS movement disorder clinics in north-west London, UK. It was required that patients taking part in this service improvement project are individuals with both idiopathic PD and mild-to-moderate psychological symptoms (primarily anxiety and depression).

This project does not meet the Health Research Authority criteria for research. It was reviewed by the Imperial College Healthcare NHS Foundation Trust Audit Office. Following relevant service improvement project registration, this work was approved by the Data Protection Office of the Imperial College Healthcare NHS Trust (PHA005; 2019), in line with the NHS Records Management Code of Practice.

The PD diagnosis was confirmed using the Queen Square Brain Bank diagnostic criteria for idiopathic PD [9]. The clinical team (five medical doctors and two Parkinson’s Specialist nurses) pre-screened eligible patients with PD at the NHS specialist clinics.

The project was introduced to PD patients with mild-to-moderate psychological symptoms who had voluntarily expressed at the specialist clinic environment that they need psychological support. The grading of psychological symptoms (absent, mild, moderate, severe, and very severe) was operationalised using clinical judgement, informed by how patients presented themselves in the movement disorders specialist NHS clinics.

Individual patients were asked to discuss the project privately with their life partner. There was no direct contact between the NHS clinical team and the partners. Patients who were interested in taking part in the project (either alone or with their partner) were assessed by the medical team at the outpatient NHS clinic environment. Patients were assessed from a mental health point of view, clinically. The assessment included a detailed review of their general medical, neurological, psychiatric, and medication history, through a structured conversation (clinical interview) focused on their mental health symptoms and experiences, feelings, thoughts and actions, general physical health and wellbeing, housing and financial circumstances, employment and training needs, social and family relationships, culture and ethnic background, gender and sexuality, use of recreational drugs or alcohol, past experiences (especially if there were any self-harm or safety issues), whether anyone depended on the individual (such as a child or elderly relative), as well as about the individual’s strengths and skills, and what would help them best, their hopes and aspirations for the future.

Clinical judgement involved the review of medical history and points made during clinical interviews and against NICE Guidelines for managing depression and anxiety disorders in adults. The focus was on anxiety and depression, and the output was qualitative: “absent” i.e. no symptoms”, “mild” i.e. intervention was not indicated, “moderate” i.e. symptoms were limiting instrumental activities of daily living, “severe” limiting self-care activities of daily living presentations and/or need for intervention in the hospital environment, and “very severe” i.e. with life-threatening consequences and/or those for whom urgent intervention was indicated.

Depression was identified as a presentation characterised by melancholic feelings of grief or unhappiness, sustained longer than 6 months. Anxiety, was identified as a presentation characterised by apprehension of danger and dread accompanied by restlessness, tension, tachycardia, and/or dyspnoea unattached to a clearly identifiable stimulus.

Patients with mild and moderate anxiety and/or depression only were considered as eligible for this service improvement project. The patients with severe and very severe anxiety and/or depression were not considered eligible for this project. The medical team also excluded patients with a history of dementia, bipolar disorder, epilepsy, psychosis, and/or other CNS comorbidity. Once eligibility was confirmed, therapists were assigned randomly to PD patients interested in getting emotional support. Eligible patients were given access to a confidential self-referral route. Through this pathway, each of the eligible patients was connected with one psychotherapist. Patients were allowed to take their usual (and new) medications for PD and any other conditions throughout their participation in this project.

The patients who did not sign up at the end of the introduction/self-referral process were consulted to contact primary care and engage through the local practice to NHS services. Feedback was sent to the NHS clinicians (including GPs) involved directly in their care.

There are several types of talking therapy, but they all involve working with a trained therapist. Different talking therapy types also suit different people. In this project, psychotherapy was provided by four accredited senior psychotherapists who had basic training in PD and PD therapeutics. The psychotherapists who took part in this project had expertise in psychosexual and relationship psychotherapy/psychodynamic counselling and psychotherapy, including eye movement desensitising and reprocessing (EMDR therapy). For the reader who is less familiar with these talking therapies, couple therapy can help people who have depression and anxiety that may be linked to problems in their relationship with their partner. Psychodynamic psychotherapy looks at how childhood experiences and thoughts an individual is not aware of (unconscious mind) can affect their thinking, feelings, relationships and behaviour today. EMDR helps the brain reprocess memories, following a traumatic event so that the negative images, emotions and physical feelings they cause, have less impact.

The project was managed by Imperial College London which is the academic institution affiliated to the Imperial College Healthcare NHS Trust. The training for psychotherapists (in PD) was provided by experienced medical doctors and researchers in PD at Imperial College London.

At registration, each participant signed a standardised confidential agreement with their therapist, in line with data protection law (EU GDPR/UK Data Protection Act 2018) and the current Ethical Framework for the Counselling Profession. With this document, patients agreed to have intervention for 12 weeks. Each patient was offered a free-of-charge 12-week therapy course (involving face-to-face weekly sessions | 50 min long for each session) to have either alone or with their partner (couple therapy). Discussing the project with the partner was not a requirement for participation; patients who did not have a partner were still eligible to take part.

In return, each participant (both patients with PD and partners, if applicable) were asked to complete an outcome measure form twice: at baseline (Week 0) and at the end of the course (Week 12 or earlier, if the course was terminated early). The Clinical Outcomes in Routine Evaluation-Outcome Measure (CORE-OM) form was used. This form has 34 statements about how they (the person who completes the form) have been doing over the last week [10, 11]. These instruments were reproduced on paper for non-commercial use and for the purposes of the service improvement project, without any change in content. The Core System Trust holds the copyright in the CORE instruments.

The following data were captured: age, number of completed sessions, compliance data/reasons for dropouts, and CORE-OM scores (at baseline and follow-up). Clinical data regarding disease duration and severity of symptoms were not recorded, as this was outside the scope of this project. Each patient was given a unique alphanumeric subject ID (provided on the blank CORE-OM form prior to clinical assessment). Completed CORE-OM forms were handed to the therapists by the patients at the end of the first and last session. Next, completed forms (coded raw data) were sent to medical team at Imperial College London by the therapists for analysis. Therapists were blinded to sub-score and total CORE-OM results.

Statistical analyses were performed using SPSS (IBM SPSS Statistics for Windows, Version 22.0. Armonk, NY: IBM Corp.). Homogeneity of variance and normal distribution were tested with Bartlett’s and Kolmogorov–Smirnov tests. Comparisons between baseline and follow-up CORE-OM scores were performed with parametric paired t-test. The level of statistical significance was set as α = 0.05.

## Results

### Recruitment, attendance and compliance rates

Overall, the self-referral process was seamless and with no significant findings/problems. The most common issue was the difficulty some individuals had in attending regular appointments due to fluctuating mobility and speech and language problems, and/or the difficulty in securing an available slot with their therapist. The second most common issue was the limited support for travel to/from the therapist office e.g. a carer being unavailable/absent to escort a patient.

**Fig. 1:**
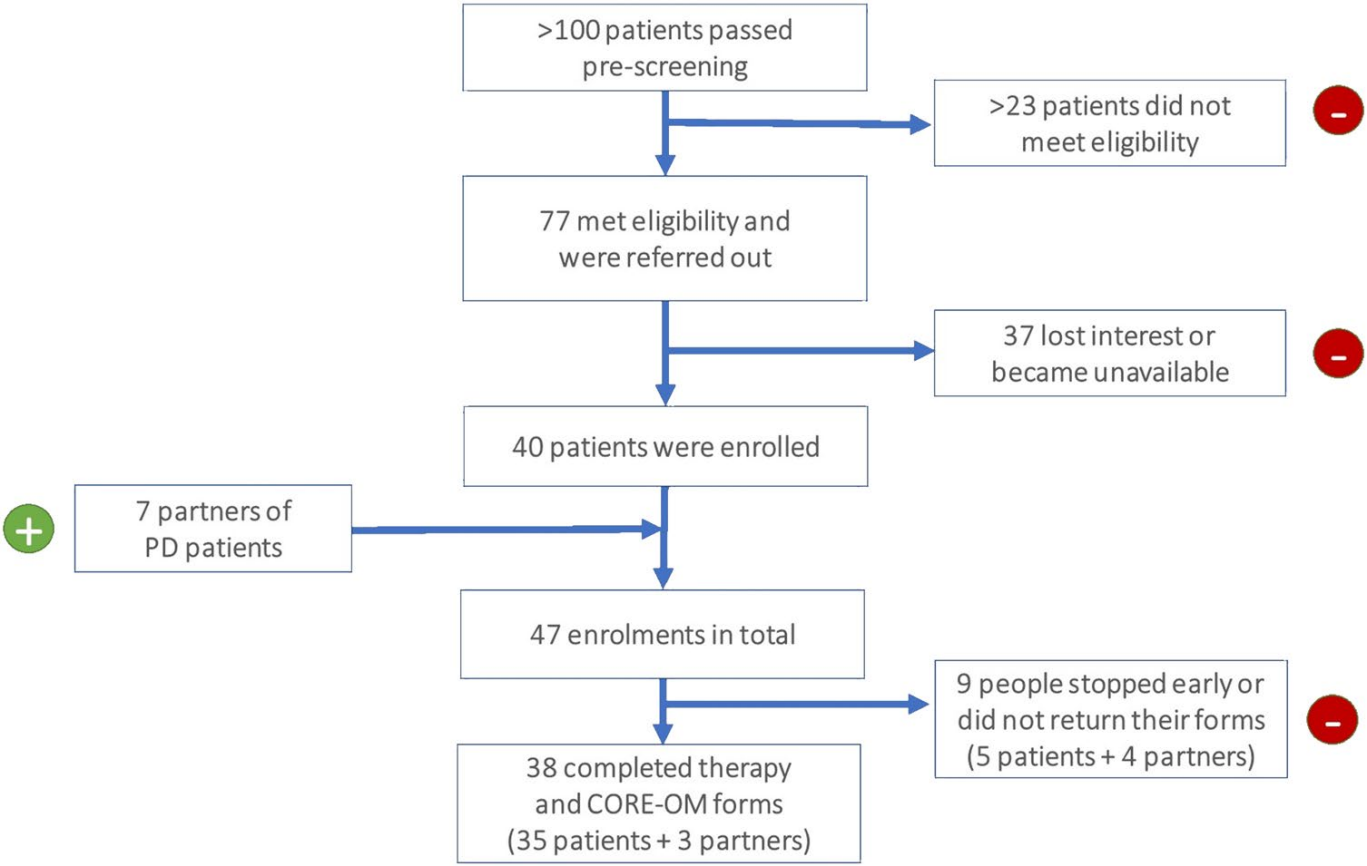
Recruitment and compliance figures

47 individuals—40 PD patients and 7 partners were enrolled in the project, aged 63.60 ± 11.37 and 67.00 ± 6.75 years old, respectively. Attendance rates were high with a mean value of 75.75% (= 9.90 sessions ± 3.02), relevant to the 12-session target. Evaluable CORE-OM data was obtained from 38 (80.85%) individuals (patients and partners); 9 (19.15%) participants stopped early or never returned their forms. See details in Fig. 1 and Table 1.

**Table 1:** Demographics, attendance and compliance rates

*N* = 47	40 Patients	7 Partners
Age	63.60 ± 11.37	67.00 ± 6.75
Attendance rates	75.75% (= 9.90 sessions ± 3.02)	
CORE-OM returns *N* = 38 (80.85%)	35 Patients (87.50%)	3 Partners (28.57%)

Values are expressed as means ± 1 standard deviation

### Primary outcome measure

Total CORE-OM scores as well as sub-scores are shown in Table 2 for the group of patients (*N* = 35). CORE-OM data collected from partners (*N* = 3) do not allow appropriate statistical comparisons and are not included in this report. PD patients showed a remarkable improvement in “wellbeing”, “problems/symptoms” and “functioning” sub-scores of CORE-OM data at the end of the 12-week period. The “risk” sub-scores were low at both time-points. There was no statistically significant change in the “risk” scores over time.

**Table 2:** CORE-OM results

	*N* = 35 (patients only)	Baseline	End-of-therapy
CORE-OM scores	Well-being	8.41 ± 3.17	5.97 ± 4.04***
	Problems/symptoms	23.43 ± 9.41	16.49 ± 9.40***
	Functioning	16.92 ± 8.58	11.51 ± 8.15***
	Risk	1.35 ± 2.23	2.09 ± 5.34^ns^
	**Total**	**50.11 ± 20.36**	36.06 ± 20.94***

Values are expressed as means ± 1 standard deviation; comparisons of means over time were sought with parametric paired *t* test

***Denotes statistical significance (*p* < 0.001)

^ns^non-statically significant (*p* > 0.05)

## Discussion

Capturing the results was important to see if intervention made any difference to the quality of life and emotional wellbeing of participants. Our findings demonstrate that patients with PD and mild-to-moderate psychological symptoms can benefit significantly from a short course of psychotherapy and counselling, through a self-referral approach, introduced at the NHS environment.

While pharmacological interventions remain central in addressing psychiatric symptoms in PD [5, 12–14], our results highlight the importance of talking therapy intervention in addressing unmet needs in patients with mild-to-moderate psychological symptoms. The observed improvement in CORE-OM “problem/symptoms” and “functioning” sub-scores demonstrate that a 12-week course of therapy is efficacious in addressing mild-to-moderate anxiety and depression measures.

Integrating talking therapy into PD therapeutics introduces a holistic approach to clinical care. In this project, patients with PD reported improvement in overall wellbeing (relevant CORE-OM scores) that further supports the above point. We appreciate that by not recording patient medication data (this was outside the scope of this project), our results may have been influenced, in theory, by the beneficial effects of CNS medication. We recognise that for decades, clinical research has been comparing psychotherapy to the effects drugs can achieve, and less about the role of psychotherapy itself alongside medications [14, 15].

Short-term psychosexual and relationship psychotherapy/psychodynamic counselling and psychotherapy support patients as a non-invasive and accessible intervention option. Both complement traditional drug therapy and medical devices approaches without posing new risks/without competing with the benefits of medicinal or surgical treatments. Based on our dataset, there was no additional harm to the participants of this project. The absence of a statistically significant change in the “risk” sub-scores highlight this point.

We propose that this project is a source of robust and informative data due to the following reasons: (i) therapists had basic knowledge of PD prior to initiating intervention; (ii) therapists had significant clinical experience and excellent communication skills, and (iii) patients were offered the opportunity to work with their partners on relationship and sexual heath (psychological) matters, the science of which overlaps with common typologies including PD.

This project was offered at no cost to NHS patients. The value proposition of this short-term intervention in PD is not discussed here, as neither analytics nor cost-effectiveness data were collected. However, it is worth noting that talking therapy is most commonly associated with private care. Insurance carriers (including national health and care systems) typically allocate limited resources for psychotherapy and counselling [16–18]. This factor independently hinders effective implementation of mental health transformation initiatives and reinforces the need to conduct research in this field.

Talking therapy as a concept, may not be well-understood, in the general population, if one is seeking support within their family of origin/in the extended family [19, 20], and not through an accredited mental health service provider. Despite any cultural and socio-economic barriers, our data support the notion that psychotherapy is an effective intervention method to reduce risks and improve clinical benefits in patients with PD who are following a traditional medical approach [5, 6]. Yet, stigma [19–21] surrounding psychotherapy may discourage self-referral and the ability of individuals to build trust with the therapist. The latter point could be, partly explained, by the common fear of individual patients being judged and fear that confidentiality may not be upheld or respected (the latter point is a finding of this project).

We tackled these issues by promoting the project openly and transparently in the NHS hospital environment by following standardised procedures and strict quality assurance measures to safeguard patient confidentiality and to ensure integrity. Moreover, we offered psychotherapy sessions free-of-charge, to all patients and partners, to increase engagement and inclusivity.

We acknowledge that the size of the population is relatively small and that we were not able to collect sufficient data from partners. The lack of distinct control groups, combined with the absence of clinical data on disease severity and medication usage question how representable this patient group is. Although cultural and socio-economic data were not collected, it is important to note that north-west London is a highly diverse, albeit geographically small urban region [22]. Additionally, we recognise that this is a project that was conducted in the north-west London ecosystem, and therefore, broad generalisations should be avoided.

## Reflections and conclusions

This report sets the background for a discussion for research in mental health within the non-motor symptoms field of PD. This dataset supports the conduct of novel research on the long-term effectiveness of talking therapy in PD, the optimal delivery methods, and the underlying reasons for stigma and hesitation of patients to engage with available NHS services. Stigma also includes hesitation from health and care professionals to promote research and service improvement projects in this field.

We conclude that a short course of talking therapy can be highly beneficial for PD patients with mild-to-moderate psychological symptoms. We propose that psychotherapy should be integrated into personalised PD treatment plans and included in large-scale clinical research proposals in the field of mental health.

## Data Availability

This dataset belongs to the Imperial College Healthcare NHS Trust. Data cannot be disclosed without permission by the ICHT Data Protection Office.

## References

[1] Reijnders JS et al (2008) A systematic review of prevalence studies of depression in Parkinson’s disease. Mov Disord 23(2):183–313. 10.1002/mds.21803 17987654 10.1002/mds.21803

[2] Dissanayaka NN et al (2010) Anxiety disorders in Parkinson’s disease: prevalence and risk factors. Mov Disord 25(7):838–845. 10.1002/mds.22833 20461800 10.1002/mds.22833

[3] Weintraub D et al (2022) The neuropsychiatry of Parkinson’s disease: advances and challenges. Lancet Neurol 21(1):89–102. 10.1016/S1474-4422(21)00330-6 34942142 10.1016/S1474-4422(21)00330-6 PMC8800169

[4] van Nuland AJM et al (2020) GABAergic changes in the thalamocortical circuit in Parkinson’s disease. Hum Brain Mapp 41(4):1017–1029. 10.1002/hbm.24857 31721369 10.1002/hbm.24857 PMC7267977

[5] Angelopoulou E et al (2023) Pharmacological and non-pharmacological treatments for depression in parkinson’s disease: an updated review. Medicina (Kaunas). 59(8):1454. 10.3390/medicina59081454 37629744 10.3390/medicina59081454 PMC10456434

[6] Luo F et al (2021) Efficacy of cognitive behavioral therapy on mood disorders, sleep, fatigue, and quality of life in Parkinson’s disease: a systematic review and meta-analysis. Front Psychiatry. 12:793804. 10.3389/fpsyt.2021.793804 34966313 10.3389/fpsyt.2021.793804 PMC8710613

[7] Dobkin RD, Menza M, Allen LA et al (2011) Cognitive-behavioral therapy for depression in Parkinson’s disease: a randomized, controlled trial. Am J Psychiatry 168(10):1066–1074. 10.1176/appi.ajp.2011.10111669 21676990 10.1176/appi.ajp.2011.10111669 PMC3186855

[8] Chaudhuri KR et al (2007) The metric properties of a novel non-motor symptoms scale for Parkinson’s disease: Results from an international pilot study. Mov Disord 22(13):1901–1911. 10.1002/mds.21596 17674410 10.1002/mds.21596

[9] Hughes AJ, Daniel SE, Kilford L, Lees AJ (1992) Accuracy of clinical diagnosis of idiopathic Parkinson’s disease: a clinico-pathological study of 100 cases. J Neurol Neurosurg Psychiatry 55(3):181–184. 10.1136/jnnp.55.3.181 1564476 10.1136/jnnp.55.3.181 PMC1014720

[10] CORE-OM form [English version] available at: https://www.coresystemtrust.org.uk/wp-content/uploads/2020/03/CORE-OM_English_fillable_optional_items_minimal_header_buttons.pdf

[11] CORE-OM form licence available at: https://www.coresystemtrust.org.uk/copyright.pdf

[12] Campagnolo M et al (2023) The pharmacological management of the behavioral aspects of Parkinson’s disease: an update. Expert Opin Pharmacother 24(15):1693–1701. 10.1080/14656566.2023.2240228 37493445 10.1080/14656566.2023.2240228

[13] Taximaimaiti R, Luo X, Wang XP (2021) Pharmacological and non-pharmacological treatments of sleep disorders in Parkinson’s disease. Curr Neuropharmacol 19(12):2233–2249. 10.2174/1570159X19666210517115706 33998990 10.2174/1570159X19666210517115706 PMC9185775

[14] Usnich T et al (2023) Depressive symptoms in Parkinson’s disease are insufficiently but more often treated than in other chronic conditions. NPJ Parkinsons Dis. 9(1):113. 10.1038/s41531-023-00551-8 37452071 10.1038/s41531-023-00551-8 PMC10349053

[15] Lake J, Turner MS (2017) Urgent need for improved mental health care and a more collaborative model of care. Perm J 21:17–024. 10.7812/TPP/17-024 28898197 10.7812/TPP/17-024 PMC5593510

[16] Mongelli F, Georgakopoulos P, Pato MT (2020) Challenges and opportunities to meet the mental health needs of underserved and disenfranchised populations in the United States. Focus (Am Psychiatr Publ) 18(1):16–24. 10.1176/appi.focus.20190028 32047393 10.1176/appi.focus.20190028 PMC7011222

[17] Arundell LL et al (2020) Advancing mental health equality: a mapping review of interventions, economic evaluations and barriers and facilitators. Syst Rev. 9(1):115. 10.1186/s13643-020-01333-6 32456670 10.1186/s13643-020-01333-6 PMC7251669

[18] Kavanagh BE et al (2023) A scoping review of the barriers and facilitators to accessing and utilising mental health services across regional, rural, and remote Australia. BMC Health Serv Res. 23(1):1060. 10.1186/s12913-023-10034-4 37794469 10.1186/s12913-023-10034-4 PMC10552307

[19] Vaishnav M et al (2023) Stigma towards mental illness in Asian nations and low-and-middle-income countries, and comparison with high-income countries: a literature review and practice implications. Indian J Psychiatry 65(10):995–1011. 10.4103/indianjpsychiatry.indianjpsychiatry_667_23 38108051 10.4103/indianjpsychiatry.indianjpsychiatry_667_23 PMC10725213

[20] Gallimore JB et al (2023) Impact of mental health stigma on help-seeking in the Caribbean: systematic review. PLoS One. 18(9):e0291307. 10.1371/journal.pone.0291307 37699044 10.1371/journal.pone.0291307 PMC10497129

[21] Kaur K (2024) “Reputation, reputation, reputation! Oh, I have lost my reputation!”; a literature review on alcohol addiction in the British Sikh and/or Punjabi community and the barriers to accessing support. Alcohol Alcohol. 59(2):agad080. 10.1093/alcalc/agad080 38016798 10.1093/alcalc/agad080

[22] Health and Care Strategy for North West London 2023. North West London Integrated Care System. Available at: https://www.nwlondonicb.nhs.uk/application/files/6016/9902/9759/NW_London_ICS_Health_and_Care_Strategy_2023.pdf

